# Metabolomics: A Viable Approach to Understand the Pathogenesis of Reactive Arthritis

**DOI:** 10.2174/0118715303309669250319083147

**Published:** 2025-04-03

**Authors:** Durgesh Dubey, Reena Kumari, Amit Singh, Pallab Shaw, Ashish Kothari, Shashi Ranjan Mani Yadav, Garima Mamgain, Shivmurat Yadav, Sandeep Kumar

**Affiliations:** 1 Department of Biological Chemistry, Center for Biomedical Research, SGPIMS Campus, Lucknow, India;; 2 Department of Medicine, Oklahoma University Health Sciences Center, Oklahoma City, USA;; 3 Department of Chemistry and Biochemistry, The University of Oklahoma, Norman, USA;; 4 All India Institute of Medical Sciences, Rishikesh, India

**Keywords:** Pathogenesis, arthritis, antigen, cytokines, spondyloarthropathy, synovitis

## Abstract

Reactive arthritis (ReA) is characterized by immune-mediated sterile synovitis brought on by an infection that enters the body through the gastrointestinal or urogenital tracts from a distance. The diseases known as seronegative spondyloarthropathy (SSA) include undifferentiated arthritis (uSpA) and reactive arthritis (ReA). Cytokines are crucial in orchestrating an effective immune response to eliminate bacterial infections, such as those seen in ReA (Reactive Arthritis) conditions. The balance between Th1 and Th2 cytokines is particularly important in determining the outcome of infections associated with ReA. TNF-α and IFN-γ are key antibacterial Th1 cytokines that promote cell-mediated immunity, essential for effective cellular responses against intracellular bacteria. In contrast, Th2 cytokines like IL-4, IL-5, IL-9, and IL-13 are more involved in generating humoral immunity and allergic responses. The mechanisms underlying the differentiation of T helper lymphocytes, which lead to a skewed cytokine secretion profile, remain unclear. Several factors, including the local inflammatory environment, IL-12 levels during T cell priming, variations among antigen-presenting cells (APCs), and antigen dose, have been suggested as potential contributors. This review will explore the critical role of metabolomics in cytokine production and its profound impact on the pathogenesis of reactive arthritis.

## 1 INTRODUCTION

Reactive arthritis (ReA) is characterized by immune-mediated sterile synovitis triggered by infections entering through the gastrointestinal or urogenital tracts. It belongs to seronegative spondyloarthropathies (SSAs), which include ReA and undifferentiated spondyloarthritis (uSpA) [1]. This group includes ankylosing spondylitis (AS), psoriatic arthritis (PsA), ReA, inflammatory bowel disease-associated arthritis (IBD-A), and uSpA, sharing features like sacroiliitis, enthesitis (the site where tendons and ligaments insert), uveitis, seronegativity for Rheumatoid factors (RF), and a strong correlation with HLA-B27 [2]. ReA is a known example of microbial involvement in inflammatory arthritis, typically developing 1-4 weeks after gastrointestinal or genitourinary infection. Symptoms include acute or subacute asymmetrical arthritis of the lower limbs of the joints with or without fever, enthesitis, dactylitis, oral ulcers, balanitis, and conjunctivitis. Formerly called Reiter’s syndrome, the term ‘ReA’ is now preferred due to its accuracy and historical context, as Hans Reiter was not the first to describe the condition—it should be attributed to Leroy-Fiessinger [3]. Briefly, ReA is characterized by the classic triad of symptoms involving the urethra, conjunctiva, and synovium. The old terminology has been replaced with ReA, partly because the urethritis characteristic could misleadingly suggest a genitourinary trigger [3]. Enterically acquired ReA is induced by a panel of bacteria, including *Salmonella typhimurium* [4], *Shigella flexineri* [5], *Compylobacter jejuni* [6], and *Yersinia enterocolitca* [7], while genitourinary acquired ReA develops due to *Chlamydia trachomatis* infection. A substantial number of patients who do not meet the diagnostic criteria for any of the currently recognized diseases within the SSA group [8] are labeled as undifferentiated spondyloarthritis (uSpA) implying that over time they would develop symptoms of well-defined members like Ankylosing spondylitis or Psoriatic arthritis which fully satisfies the requirements of the European Spondyloarthropathy Study Group (ESSG), such as inflammatory low backache or asymmetrical lower limb arthritis, with at least one of the following features: buttock pain, enthesitis, radiological evidence of sacroiliitis, or a positive family history [9]. Patients of this group closely resemble ReA yet without any preceding history of enteric or genitourinary infection. The evidence for microbial involvement in uSpA has been reported despite the absence of symptomatic diarrhea like in patients with enteric/genitourinary acquired ReA. Gut inflammation of varying degrees has been identified in intestinal biopsy specimens [10], while *Chlamydia* DNA has been found in the synovial fluid and synovial membrane of individuals with ReA and uSpA [11]. Serum IgA antibodies against enteric bacteria *S. Typhimurium, S. flexneri,* and *Y. enterocolitis* has been found to be elevated in patients with ReA as well as with uSpA [12]. The proliferation of synovial fluid mononuclear cells (SFMCs) to whole enteric bacterial extract and purified membrane protein of *Salmonella* constituents in patients with ReA and uSpA [13, 14] also indicate that uSpA has a similar clinical picture and immune response to microbial trigger despite the lack of preceding symptomatic infection.

## 2 CLINICAL PRESENTATION OF PATIENTS WITH REA

The clinical symptoms of ReA are the same regardless of whether it is acquired enterically or venereally.. Though post-enteric ReA is equally prevalent in males and females aged 20 to 40, males are more commonly impacted in cases of venerally acquired ReA, with a male-to-female ratio reported at 9:1 [15]. Days to several weeks following the triggering infection, the disease manifests as peripheral monoarthritis or asymmetric oligoarthritis, which may be accompanied by fatigue, fever, and malaise [16]. Lower limb joints (knee, ankle, and foot) are affected asymmetrically, mimicking a picture of septic arthritis. ReA typically lasts four to five months, although 15% to 30% of individuals may experience chronic illness [17]. Axial joint involvement, including the hips and spine (cervical, dorsal, or lumbar), causes pain and limited movement. Asymmetric sacroiliitis is radiologically evident in 14–49% of ReA patients, affecting one-third of urogenital and one-tenth of enteric ReA cases [18].

Enthesitis and dactylitis are common features of ReA shared with other spondyloarthropathies. Enthesitis, inflammation where ligaments and tendons attach to the bone, often affects the plantar aponeurosis and Achilles tendon, which attach to the calcaneus, causing pain in the heel and walking difficulty. Dactylitis, seen in 16% of ReA cases, presents as diffuse swelling of an entire finger or toe, known as a “sausage digit” [18, 19].

Conjunctivitis with mucopurulent discharge is the most common ophthalmologic symptom evident in around 35% of ReA patients. Dermatologic manifestations include circinate balanitis, and keratoderma blennorrhagica, occurring in 20–40% and 5–30% of patients, respectively [19]. Painless oral ulcers are also seen in patients with ReA. Erythema nodosum is a known complication of *Yersinia*-triggered ReA, marked by painful nodules mainly appearing on the extensor surfaces of the arms and legs [17, 20]. Baseline clinical characteristics used to diagnose ReA include elevated inflammatory markers such as erythrocyte sedimentation rate (ESR), C-reactive protein (CRP), as well as total blood count and urine analysis. During the acute stage, individuals may also exhibit other markers of the inflammatory response, such as thrombocytosis or leukocytosis. Antinuclear antibody (ANA) and radiofrequency (RF) are evaluated to distinguish this arthritis from others. ReA is more likely in cases where the HLA-B27 test is positive, although this test lacks both sensitivity and specificity for the condition. Therefore, it is not used for diagnosis of ReA despite its strong association with the disease. Since *Salmonella* and *Yersinia* persist in the gut even after several weeks after the resolution of diarrhea, the stool culture technique can be used to diagnose gastrointestinal infection. For diagnosing genitourinary infections, nucleic acid amplification methods like ligase chain reaction (LCR) and PCR are preferred. These techniques use urine and swab samples from the urethra or vagina, as *Chlamydiae* are difficult to culture [3].

## 3 EPIDEMIOLOGY

The absence of a gold standard diagnostic test for ReA complicates interpreting its incidence and prevalence, which are influenced by genetic differences, environmental conditions, geographic distribution and the prevalence of causative pathogens [21].

Reports based on the questionnaire survey and examination, following gastrointestinal infection with *Salmonella*, *Campylobacter*, *Yersinia* and *Shigella*, clinical ReA have been seen in 7-12% of subjects [22-25]. Most of the epidemiological data come from Europe. The estimated prevalence of ReA is around 0.1% [26, 27]. Incidence rates vary, with post-venereal ReA occurring at a rate of 4.6 to 13 cases per 100,000 people and post-enteric ReA occurring at a rate of 5 to 14 cases per 100,000 people [27, 28]. In the European population, the prevalence of ReA is around 9/100,000 in most countries: Norway 9.6/100,000 [27]; Finland 10/100,000 [29]; and 9.3/100,000 in the Czech Republic [30], while in Southern Sweden it is considerably higher at 28/100,000 [31]. The uSpA and ReA, who fulfilled ESSG criteria for spondyloarthropathy, constituted 14% and 1.2% of 1379 patients, respectively, according to a Spanish registry-based study of one-year duration which involved 12 reference centers across the country [32]. Based on a questionnaire survey, the prevalence of both diseases was 0.09% calculated among 2155 respondent subjects in a central region of Italy [33]. The estimated data from Berlin showed that the prevalence of uSpA and ReA in Germany was 0.6% and 0.1% [26]. Previous studies reported varying prevalence rates for ReA: a study from Russia found a prevalence of 0.6%, while a study from Alaska reported a prevalence of 0.1% [34, 35]. In Spain, two registry-based studies reported that ReA was present in 1.2% to 1.4% of SSA patients [32]. Conversely, in Argentina, ReA was identified in 25 out of 402 SSA patients, which accounts for 6.2% [36]. Regarding the microbial-specific ReA, *Y. enterocolitica* is frequently used to induce ReA in European nations, particularly in Scandinavian nations like Norway and Finland [37]. In Finland, the incidence rate of campylobacter-induced ReA was 4.3 per 100,000 cases [23]. According to reports from Norway, the annual incidence of ReA due to chlamydia is 4.6 cases per 100,000 people. In contrast, ReA triggered by intestinal bacteria occurs at a rate of 5 cases per 100,000 individuals each year [27]. The causal pathogen was found in 29/52 (56%) of the ReA patients from several rheumatology clinics located throughout Berlin, Germany, by using tool culture and sera antibodies to various microbes, agglutination, ELISA and Immunofluorescence assay. From these, 17 were gastrointestinal infection (33% *Salmonella* and 18% *Yersinia*) induced ReA [38]. The clinical picture of possible ReA in 74 patients (with no symptomatic history of previous infection or uSpA), *Yersinia* (19%), *Salmonella* (12%) and *C. trachomatis* (16%) were identified as trigger pathogens in the same study [38].

In the USA, the incidence rate of ReA was estimated as 0.6-3.1/100,000 cases. According to a study from Oregon and Minnesota, USA, 6379 patients were diagnosed with a culture-confirmed enteric infection from *Campylobacter jejuni*, *E. coli* O157, *S. tymphimurium*, *S. flexenri* and *Y. enterocolitica.* The incidence was highest for *Compylobacter* (2.1/100,000), while for *Salmonella* it was 1.4/100,000 [39-41].

## 4 AETIOLOGY

Despite extensive research, the pathophysiology of ReA remains unclear. It is an infectious disease triggered by an immunological response to specific infections in genetically susceptible hosts, the most important predisposing factor being HLA-B27. The list of causative microorganisms continues to grow.

### 4.1 Genetic Factors

A significant genetic contribution to the development of ReA is shown by HLA-B27. The association with AS is particularly clear, as 94% of patients test positive for HLA-B27, in contrast to just 9% of the general population [42]. Later, HLA-B27 was connected to additional members of the SSA family, such as ReA (Brewerton) [43], IBD-A [44], and PsA [45]. The prevalence of the disease is five times greater in HLA-B27-positive individuals compared to the general population and ten times higher in HLA-B27 relatives of patients with ReA, highlighting the strong correlation between HLA-B27 and ReA [46]. Transgenic animals offer direct evidence of this correlation. HLA-B27 transgenic rats develop a multi-system illness that exhibits many features similar to human disorders associated with HLA-B27 [47]. Additionally, symptoms often emerge when transitioning from a pathogen-free environment to more typical settings [48]. The susceptibility to illness in transgenic mice is significantly associated with the level of HLA-B27 expression and the number of gene copies [49]. Moreover, the presence of HLA-B27+ T cells [50] and bone marrow cells [51] is essential, aligning with the characteristics of immune-mediated diseases.

HLA-B27 is an MHC class I protein consisting of β2-microglobulin (β2m, 12 kDa) non-covalently linked to a glycosylated heavy chain (45 kDa) [52]. The class I heavy chain includes a transmembrane region, a cytoplasmic tail, and three extracellular domains: α1 (N-terminal), α2, and α3. The α1 and α2 domains create a highly polymorphic peptide-binding cleft with six pockets (A, B, C, D, E, and F), supported by two antiparallel α-helices on a base of eight antiparallel β-strands [53-55]. The defining feature of HLA-B27 is its B pocket, with cysteine residue at the mouth and glutamic acid at the base, exhibiting high specificity for an arginine side chain at the second position of bound peptides. This arrangement is consistent across all HLA-B27 subtypes associated with disease [56]. A unique feature of the HLA-B27 molecule is the unpaired cysteine-67 residue in the extracellular α1 domain that facilitates disulfide bonding between the two heavy chains to form a “homodimer” [57-59]. These inherent “defects” suggest abnormal cell biology in HLA-B27, potentially contributing to pathogenic processes. To date, over 105 molecular subtypes of HLA-B27 have been recognized [60]. Among these, the most common subtypes— HLA-B*2702, B*2704, B*2705, and B*2707—are strongly associated with an increased risk of developing spondyloarthritis. The most common variety among them, B*2705, is linked to spondylitis over a wide range of racial and geographic divides. Even though HLA-B27 has continued to be the subject of much research, the precise mechanism by which HLA-B27 influences illness susceptibility is still unknown. Furthermore, it is still unclear why specific organ systems—like the joint, spine, stomach, and eyes—are attacked by HLA-B27-associated disorders but not others.

The arthritogenic peptide model proposes that ReA is triggered by cytotoxic T cells responding to a joint-specific peptide presented by HLA-B27 subtypes. Normally, the peptide's low concentration prevents T-cell recognition. During infection, bacterial proteins mimicking the peptide sensitize T cells, enabling recognition at low levels and initiating an autoimmune response against self-antigens, leading to SpA.

The arthritogenic peptide model suggests that ReA is triggered by cytotoxic T cells responding to a joint-specific peptide presented by all HLA-B27 subtypes. The fundamental function of HLA-B27 as a class I antigen in the presentation of antigenic peptides to cytotoxic lymphocytes serves as the foundation for this notion. However, in “normal” conditions, the peptide’s low concentration supply prevents T cell recognition. During infection, bacterial proteins with sequence motifs resembling the arthritogenic peptide sensitize T cells, enabling recognition at low levels and initiating an autoimmune response against self-antigens, leading to loss of self-tolerance and ultimately SpA, resulting from an autoimmune response driven by T cells targeting self-antigens [61]. Previous studies suggest that CD4^+^ T cells might also be involved in class I-restricted immune recognition [62]. Consequently, SpA may involve a CD8^+^ or CD4^+^ T cell response that is restricted by HLA-B27 and directed against microbial or self-peptides.

According to the arthritogenic peptide theory, HLA-B27 must selectively bind to distinct peptide epitopes with heightened precision. Subtype polymorphisms affect how HLAB27 presents peptides inside peptide-binding grooves. Except for their distinctive conserved B pocket, HLA-B27 subtypes differ at several critical locations within the peptide binding groove [54]. Evidence supporting this idea includes the identification of HLA-B27-restricted peptides derived from *Chlamydia trachomatis* [63] and the resemblance between endogenous B27 peptides and environmental antigens [64, 65]. These days, arthritic symptoms are increasingly used to distinguish SpA from other types of arthritis [66]. In SpA, studies are investigating the potential role of cartilage proteoglycans, such as versican and aggrecan, along with the link protein, as possible autoantigens [67]. In BALB/C-B27 transgenic mice, It has been demonstrated that the characteristic inflammation of SpA, particularly tenosynovitis, is triggered by HLA-B27-restricted epitopes derived from human aggrecan [68].

Abnormal processing, especially concerning the folding of the HLA-B27 heavy chain, has garnered significant attention. Normally, cell surface HLA-B27 comprises a peptide produced in the endoplasmic reticulum and a heavy chain linked to P2IT1 [69]. Recent descriptions have indicated that newly synthesized HLA-B27 folds slowly, associates with β2m slowly, and tends to misfold because of its distinct B pocket design [70]. A pro-inflammatory “unfolded protein response” could be triggered by the buildup of misfolded HLA B27 protein, and this could potentially contribute to the pathophysiology of diseases linked to HLA-B27 [71]. According to a recent investigation, human CD4^+^ T lymphocytes isolated from SpA patients recognize HLA-B27 itself [72]. Additionally, neither MHC II nor the transporter linked to antigen processing (TAP) are involved in this unusual recognition. This recognition involves a range of molecules, including free HLA-B27 heavy-chain monomers, non-conventional homodimers, and conventional HLA-B27 heterodimers. The HLA-B27 restricted TCR transgenic mice (GRb) produce functional CD4^+^ and CD8^+^ T cell responses, as demonstrated earlier by Roddis *et al*. [71, 73], which lends credence to these findings. Studies on adoptive transfers have demonstrated that CD4^+^ T cells have a more significant role in the elicitation of arthritis than CD8^+^ T cells [51]. Collectively, our results point to a role for atypical MHC l-restricted CD4^+^ T cell identification in the pathophysiology of SpA.

A theory known as 'auto-display' of HLA-B27 has been proposed to explain its role in SpA [74]. This hypothesis suggests that heavy chains lacking peptides, or β2m-free heavy chains, facilitate a transition from a helical to a coiled structure between the α2 and α3 domains. This transition facilitates the rotation of backbone angles around residues 167/168, enabling the molecule to fit into the peptide binding cleft more effectively. While experimental evidence for the existence of such assemblies is limited, the auto-display of HLA-B27—whether within or between HLA-B27 molecules—could underpin self-sustaining inflammatory and immunological activation. A different theory proposes the deposition of β2m [75]. This hypothesis accounts for the presence of P2IT1 in synovial fluid as amyloid aggregates. It is based on the observation that disease-associated HLA-B27 subsets have a higher rate of β2m dissociation from the HLA-B27 complex compared to non-associated subsets, leading to an immune response against these aggregates. With this specific concept, HLA-B27 itself seems to be accidental rather than crucial to the disease mechanism. Furthermore, research on transgenic animals demonstrates the opposite conclusion, namely that spontaneous inflammatory arthritis can be caused solely by β2m loss without HLA-B27 expression [76]. The pathogenesis of ReA may be influenced by the intracellular persistence of arthritogenic microorganisms. This suggests that in susceptible individuals, abnormal host-microbe interactions can lead to the ineffective clearance of arthritogenic bacteria, their fragments, or both following the initial infection. Supporting this idea, previous research [71] found that HLA-B27 expression decreases the invasion of arthritogenic bacteria into transfected L cells. Beyond invasion, HLA-B27 also significantly reduces *Salmonella* enteritidis's ability to be eliminated from transfected human monocytic cells [77].

Furthermore, compared to control groups, HLA-B27-positive monocytes exhibit a lower efficiency in killing Salmonella *in vitro*. Additionally, they show elevated levels of TNFα and interleukin 10 (IL-10), with IL-10 being increased to a lesser extent [78, 79]. These findings suggest that HLA-B27-associated modulation of cytokine response profiles may influence the pathophysiology of the disease. According to this theory, HLA-B27 may serve purposes other than just presenting antigens. However, further research has shown that HLA-B27 peptide presentation is not influenced by arthritogenic bacteria [80], and that Salmonella invasion and persistence in fibroblasts are not impacted by whether the cells are from HLA-B27 positive or negative individuals [81]. Given that both factors have a significant impact on the outcome, the varied results could be a reflection of the characteristics of the various cells and bacterial strains used in these investigations. This idea considers the role of genetic vulnerability and triggering bacteria in the pathogenesis of ReA, and although the results are still contentious, it is worth looking into further.

### 4.2 Microbial Factors

As previously noted, ReA is a well-documented joint inflammation that occurs following infections with certain Gram-negative bacteria; however, the exact initiating infection in other SpA family disorders is less evident [82]. When placed in a germ-free environment, HLA-B27 transgenic rats do not develop inflammatory disease in their joints or intestines. Furthermore, the idea that these microorganisms play a crucial role in the pathophysiology of HLA-B27-related disease is reinforced by the induction of arthritis following the reintroduction of commensal gut flora [83].

#### 4.2.1 Presence of Bacteria or their Components in the Joints as Evidence

Several methods have been employed to demonstrate that *chlamydia* is the most common causal agent of ReA in joints. In synovial tissues and peripheral blood, chlamydia DNA, mRNA, rRNA, and entire, non-culturable, living cells have been detected [84-89]. Chlamydial mRNA and rRNA are nucleic acids with a half-life of only minutes in tissues. Their presence indicates ongoing transcription, which is associated with active bacterial replication [85]. Additionally, an inverse relationship has been observed between the presence of Chlamydial DNA in the joint and the proliferation of Chlamydia-specific lymphocytes in the synovial tissue [90], raises the possibility that the persistence of bacteria is due to a compromised T-cell response, which may be connected to HLA-B27 expression.

Attempts to grow *Salmonella* or *Yersinia* from the affected joints have been unsuccessful. However, DNA from *Yersinia*, *Shigella*, or *Campylobacter* has been detected in some studies [18, 91-94]. Antigens associated with these microorganisms have been detected in patient samples of synovial tissue or synovial fluid [92, 93]. ReA patients' joints have been shown to include bacterial products such as Yersinia LipoPolySaccharide (LPS), *Salmonella*, *Shigella* [91, 92, 95], *Chlamydia trachomatis* [96], and the *Yersinia* proteins YadA [93] and heat shock protein [97]. Additionally, *Salmonella* LPS was detected in synovial tissue two years after the initial infection [91]. Heat shock proteins from *Yersinia* and LPS have been identified in synovial fluid and peripheral blood cells for up to four years in ReA patients [97]. High concentrations of antibodies have also been present in patient serum samples for several months [98].

All the research discussed indicates that bacteria or their byproducts persist in the host long after the initial infection. Through blood vessels, these bacteria or their components can enter the synovial tissues either as whole organisms circulating in the blood (bacteraemia) or as parts of immune complexes within cells [18, 91, 93]. It has been proposed that monocytes, including macrophages, might serve as reservoirs or transporters of bacteria to the synovial tissue [18]. This hypothesis is supported by the observation that macrophages, which constitute about 50% of the lining layer's cells, originate from peripheral blood [99], a key feature of the synovium. When tissue macrophages are derived from blood monocytes, bacteria or their components phagocytosed by these cells at the site of infection (mucosa) or in the blood are likely to reach the joints, with the amount dependent on recruitment rates. More people would be recruited by joints that are large or that have some degree of inflammation from mechanical stress. Therefore, this mechanism may explain why massive lower limb joints are preferentially involved in ReA [100].

This idea is further supported by experimental data, which show that intracellular bacterial fragments within mononuclear cells from peripheral blood are especially prone to adhering to synovial high endothelial venules and migrating through the endothelial cell monolayer [101]. Furthermore, P-selectin, a protein crucial for homing to the synovium, can be expressed on cultivated endothelium cells by bacteria consuming monocytes [102]. Despite these findings indicating that monocytes/macrophages function as carriers of bacterial antigens to the joints, direct evidence for this remains lacking.

It is also possible that these bacteria exist in an extra-articular location, such as in the digestive system's mucosal membranes and/or lymphocytic tissues, and that monocytes bring them to the joint on repeat [97, 101, 103]. A recent study [104] provided a dynamic picture of *Salmonella* and *Yersinia* invasion, degradation, and persistence in synovial fluid. This study showed that bacterial infection of synovial fluid starts with the adherence of the bacteria to the membrane of synovial cells, followed by the uptake of intact bacteria into the cells. After a few hours of infection, the bacteria exhibited very low metabolic activity and began a process leading to the complete elimination of their cytoplasm. This may provide only a partial explanation for the clinical aspect of frequent recurrences observed in some ReA patients.

#### 4.2.2 Common Features of Triggering Bacteria

**Fig. (1):**
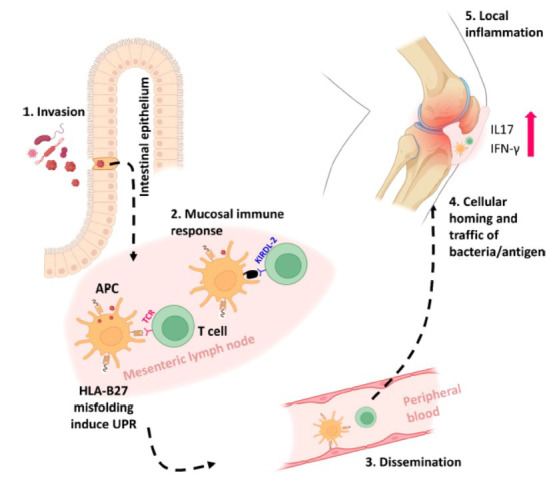
A summary of the pathogenesis of ReA. (1) Pathogenic bacteria adhere to and infiltrate the intestinal epithelium. The antigen-presenting cell (APC) HLA-B27 on macrophages may contribute to bacterial persistence. (2) APCs may use HLA-B27 to present arthritogenic peptides to CD8^+^ T cells, or HLA-B27 itself may be recognized by the killer immunoglobulin receptor (KIR)3DL2 on CD4^+^ T cells in the mesenteric lymph node (MLN). Additionally, misfolded HLA-B27 triggers an unfolded protein response (UPR). (3–4) T cells and APCs carrying bacterial antigens or inactive bacteria travel through peripheral blood and eventually reach the knee joint. (5) T cells and APCs from the gut produce IFN-γ and IL-17, which trigger an immune response, recruit other cells, and activate mesenchymal cells in the target joint, thereby enhancing and sustaining inflammation.

Several factors must be considered when analyzing the development of ReA, including the host immune response, the persistence of causative microbial antigens in the joints, environmental and genetic factors, and the presence of the HLA-B27 molecule (Fig. **1**). Certain physiologic characteristics are shared by the ReA-triggering bacteria, including their ability to exist intracellularly, the importance of LPS in their outer membrane, and their ability to infect mucosal surfaces [105]. The reason why various bacteria can exhibit similar clinical presentations and be associated with HLA-B27 remains unclear. To better understand the pathophysiology, it is essential to identify the immunodominant antigens of these bacteria [106]. It is currently unknown what antigens all bacteria have in common.

 Four criteria have been proposed for identifying potential immunodominant antigens: cross-reactivity between bacterial and human antigens, expression in the synovial environment, conservation across multiple bacterial species, and the ability of bacteria to evade the immune system. Despite extensive searches over the years for a critical arthritogenic antigen, none have been identified to date. The T-cell response in ReA appears to be primarily focused on bacterial heat shock protein (hsp)60, and autoimmunity has been linked to cross-reactivity against autologous hsp60 [107]. But T cells that are specific to Chlamydia's hsp60 are unable to respond with enterobacteria's hsp60 [108]. Yersinia-induced ReA has also led to the identification of alternative options among immunodominant antigens, such as ribosomal protein L23 and a cationic 19-kDa urease B subunit.

The idea that antigenic mimicry between evolutionarily conserved epitopes may promote autoimmunity in ReA is supported by the high degree of conservation of the urease component among bacteria [109]. Bacterial LPS plays a crucial role in the pathogenesis of ReA due to its significant impact on bacterial virulence and its ability to modulate the immune system rather than directly triggering autoimmunity. In patients with Salmonella-induced ReA, the persistent humoral immune response and intra-articular antibodies are primarily directed against LPS [98].

Additionally, LPS in synovial tissue can activate a range of inflammatory cytokines and strongly stimulate macrophages, primarily through the nuclear factor-kappa B (NF-κB) pathway [110, 111]. LPS also enhances the production of IL-8, a chemokine that attracts and activates polymorphonuclear leukocytes from chondrocytes [112], promotes the chemokine monocyte chemotactic protein, and reduces the expression of C5aR on monocytes [85]. Due to these modifications, leukocytes are drawn into the synovium, activated macrophages remain there, and chronic inflammation ensues. Since bacterial DNA has more nonmethylated CpG patterns than mammalian DNA, it can strongly excite macrophages and monocytes, which may also be a factor in inflammation. A study shows that an experimental intra-articular injection of bacterial DNA is enough to induce arthritis in mice as supported by a theory [110].

## 5 INTERACTION OF BACTERIA AND HOST IN REA

### 5.1 Humoral Immune Response

While antibodies specific to microorganisms can be useful in diagnosis, the immunopathogenesis and MHC association of this illness cannot be explained by the humoral immune response. Nevertheless, it might correlate with the illness's severity and prognosis [113]. Salmonella-induced ReA has been shown to generate antibodies of the IgM, IgG, and IgA classes [98]. IgA is the primary humoral response, particularly in enteric ReA, suggesting long-term stimulation of the intestinal mucosa. The endurance of antibodies may signify the endurance of bacterial antigens in individuals with arthritis, hence implying a compromised cellular immunity [114]. Analysis of B cells isolated from lymphoid infiltrates in the synovial tissue of ReA patients indicates that B cell development is dependent on T cells and driven by antigen exposure [115]. However, the possibility that ReA patients would not have any antibody responses suggests that they are only marginally involved in the pathophysiology of synovitis [114].

### 5.2 Cellular Immune Response

T cells are essential to the pathophysiology of ReA [18], and synovial fluid from ReA patients has been shown to include antigen-specific CD4^+^ and CD8^+^ T cells [116]. Presenting peptides to cytotoxic T cells is the role of HLA-B27. Fig. (**2**) shows how HLA-B27-restricted cytotoxic T-cell clones can combat bacterial antigens [117-119]. Additionally, blood and synovial fluid from ReA patients contain multiple expansions of T lymphocytes, most notably in the synovial CD8^+^ compartment, where these expansions often show both activation and memory markers [120]. This suggests that CD8^+^ T cells restricted by HLA-B27 might be detrimental. However, research using HLA-B27 transgenic mice lacking β2m— which have extremely low levels of CD8+ T cells yet still develop inflammatory disease— suggests that HLA-B27's role in joint disease may extend beyond merely serving as a restriction element for CD8^+^ T cells [121, 122].

Moreover, the TAP-1 gene, which is essential for loading peptides onto MHC class I molecules, is not required for disease development in these mice [121]. Additionally, adoptive transfer experiments have demonstrated that CD4^+^ T cells are more effective than CD8^+^ T cells in propagating inflammatory disease when transplanted into nude HLA-B27+ transgenic rats [51]. This suggests that CD4^+^ T cells may play a more prominent role in the pathophysiology of ReA compared to CD8^+^ T cells. Early studies showed that HLA class II-restricted CD4^+^ responses to triggering organisms were insufficient to prevent spontaneous disease in MHC-II negative HLA-B27 transgenic mice [123, 124] have led to recent proposals [125], suggesting that MHC II molecules may not be necessary for disease development. This implies that CD4^+^ T cell recognition of HLA-B27 might significantly influence the pathophysiology of the disease, as illustrated in Fig. (**2**).

Numerous studies have focused on examining homodimers formed by free HLA-B27 heavy chains that are not bound to β2m [57, 58, 126]. These homodimers have been detected on the surface of human lymphoid cell lines [57, 58, 55, 56] and in HLA-B27-positive SpA patients [127]. Some theories propose that the formation of homodimers either mimics MHC II molecules, prompting recognition by CD4^+^ T cells, or allows the binding of a different set of antigenic peptides [59]. Beyond their traditional role in antigen presentation, members of the killer immunoglobulin receptor and leukocyte immunoglobulin-like receptor families can also recognize various forms of HLA-B27, including classic or free heavy-chain monomers and homodimers [128]. Consequently, it has been suggested that HLA-B27 interacts with natural killer cells; however, the exact role of this interaction in disease pathogenesis remains unclear. HLA-B27 serves as a ligand for paired Ig-like receptors on natural killer cells. The evidence indicates that both CD4^+^ and CD8^+^ T cells may be crucial to the pathophysiology of ReA, though recent attention has focused more on CD4^+^ T cells' recognition of HLA-B27 homodimers.

### 5.3 Cytokines

While ReA generally follows a benign course in most patients, up to 15% of cases can progress to chronic arthritis lasting more than a year [129]. As previously noted, bacterial antigens have been detected in synovial tissues [18, 91, 92], and in some ReA patients, these antigens have been present in synovial fluid and peripheral blood for up to four years [97]. Evidence of a T cell response specific to synovial antigens [118, 119] suggests that the immune response may be driven locally by persistent bacteria. Additionally, the high levels of antibodies observed in the serum of ReA patients further indicate ongoing bacterial presence [98].

The reason why the immune system fails to eradicate these bacteria remains unclear, although HLA-B27 might influence bacterial invasion and persistence, potentially leading to an impaired immune response [130]. However, it is known that appropriate cytokine secretion is crucial for an effective immune response against bacterial infections [131]. Th1 and Th2 cytokines are especially important in defending against intracellular microorganisms. Thus, the balance between Th1 and Th2 responses has been suggested as a potential predictor for the outcome of bacterial infections associated with ReA [132]. Th1 cytokines, such as TNF-α and IFN-γ, are potent antibacterial agents that enhance cell-mediated immunity, essential for combating intracellular bacteria. In contrast, Th2 cytokines like IL-4, IL-5, IL-9, and IL-13 are more involved in humoral immunity and allergic reactions, and Th2 cytokine production can inhibit the effects of Th1 cytokines [133, 134]. The dominant cytokine secretion pattern in the synovial fluid of ReA patients remains a topic of debate.

A recent study [135] confirmed that individuals with Yersinia-induced ReA exhibit a strong Th1 cytokine pattern in their synovial fluid, consistent with earlier findings [136]. The high levels of TNF-α and IFN-γ observed in chronic ReA support the use of anti-TNF-α therapy, which has shown promising results, particularly for chronic ReA patients [105]. Conversely, reduced Th1 (TNF-α) cytokine production has been associated with prolonged disease duration [137]. Additionally, research suggests that Th2-associated cytokines, such as IL-4, are abundant in the synovial tissues of ReA patients [131, 138]. This presence of Th2 cytokines may hinder pathogen elimination and contribute to disease persistence. Furthermore, the presence of anti-inflammatory Th2 cytokines could help manage joint inflammation, as many ReA patients eventually experience disease resolution, though it often takes about a year [129]. Supporting this, studies in animal models have shown that IL-4 can reduce joint inflammation [139]. Reconciling the variability in cytokine profiles observed in synovial tissues across studies is challenging and may be influenced by factors such as patient selection, sampling methods, or *in vitro* processing. The underlying mechanisms driving T helper lymphocyte differentiation and the resulting cytokine profile remain unclear, though factors such as the local inflammatory environment, IL-12 levels during T cell priming, differences in antigen-presenting cells (APCs), and antigen dosage have been suggested as potential contributors [134, 140, 141]. Increased levels of IL-10 and TGF-β production have been observed in the synovial membrane of ReA patients, suggesting that another subset of CD4^+^ T cells producing these cytokines might have a regulatory role. It remains unclear whether this is a common phenomenon and how it affects the balance between Th1 and Th2 responses [142].

## 6 METABOLIC ASPECT OF INFLAMMATION

The classic cardinal symptoms of inflammation—heat, redness, swelling, and pain—characterize an acute inflammatory response [143]. In experimental settings, the timing of edema, leukocyte accumulation, and the presence of monocytes and macrophages is well-documented. Resolution of inflammation is facilitated by the release of local mediators that limit further leukocyte recruitment in self-limiting inflammatory reactions [144]. It is widely believed that an overproduction of pro-inflammatory mediators contributes to the transition from acute to chronic inflammation [145].

### 6.1 Metabolic Consequences of Inflammation

The complex progression of inflammatory reactions is influenced by a variety of factors. Microbiological, immunological, and toxic substances can all initiate the inflammatory response by activating a range of humoral and cellular mediators. In the early stages of inflammation, large amounts of interleukins and lipid mediators are released, which play a key role in the pathophysiology of organ dysfunction [146, 147]. During inflammation, membrane phospholipids release arachidonic acid (AA), which is then converted into prostaglandins and leukotrienes. Various strategies have been employed to manage the overproduction of lipid mediators, including blocking lipoxygenase and cyclooxygenase pathways, inhibiting phospholipase A2 (which releases AA), and developing receptor antagonists to block leukotrienes and platelet-activating factor [146]. Certain agents continue to protect against various inflammatory conditions like asthma, RA, and septic organ failure, whereas others do not. Long-chain omega-3 fatty acids, such as eicosapentaenoic acid (EPA), have been shown to reduce cardiovascular morbidity when incorporated into the diet [148]. During inflammation, EPA competes with arachidonic acid (AA) for enzymatic metabolism, leading to the production of less chemotactic and inflammatory derivatives [146]. When studying inflammation, it is important to consider the various factors in the inflammatory environment that may contribute to pathological conditions. Hypoxia is commonly observed in inflammatory settings, including wounds, cancer, bacterial infections, and autoimmune diseases [149, 150]. Increased hypoxia at the inflammatory site is associated with worse disease outcomes, such as more severe synovitis in RA and PsA [151].

In pathogenic conditions, tissue oxygen levels can drop from as low as 0.5% to about 2.5%, whereas normal physiological oxygen levels range from 5-12% (compared to 21% atmospheric oxygen). Local hypoxia may occur when inflammatory tissues obstruct blood vessels or when the available blood supply is insufficient to meet the increased cellular demands due to the proliferation or infiltration of inflammatory cells [152].

Furthermore, decreased blood flow into the inflammatory location could be caused by circulating phagocytes blocking blood arteries [153]. When normal tissue structures like the cornea or synovium are inadequately perfused, they might contribute to hypoxia. By changing blood vessel pressure, resulting in vessel blockage and lengthening blood vessel distances, tissue modification brought on by inflammation can cause hypoxia [154, 155]. There is substantial evidence indicating that the inflammatory synovium is a hypoxic environment. Similar to tumor environments, which are known for their hypoxia and significant angiogenesis, the inflammatory tissue also exhibits a high demand for increased oxygen supply [156]. Synovial fluid from RA patients had lower oxygen tensions than that of individuals with osteoarthritis and severe joint injuries, according to direct measurements of oxygen levels in the fluid [157].

Cells respond to changes in ambient oxygen through a sophisticated oxygen-sensing system. Hypoxia-inducible factor (HIF), a transcription factor usually marked for degradation under oxygen-rich conditions, becomes stabilized when environmental oxygen levels decrease. HIF consists of two subunits, α and β, with the stability of the α subunit being oxygen-dependent. Under hypoxic conditions, HIF-α dimerizes with HIF-β, avoiding degradation [158]. Consequently, hypoxia is associated with increased HIF expression, which has been observed in autoimmune diseases such as multiple sclerosis and rheumatoid arthritis [159-161]. HIF is well known for its critical role in inflammation; for example, reduced HIF-1α in macrophages is associated with decreased bacterial invasiveness, motility, aggregation, and survival [162]. Hypoxia and HIF stabilization significantly impact cellular metabolism by increasing the expression of glycolytic enzymes, promoting glycolysis over oxidative phosphorylation. This allows ATP production to continue even with limited oxygen, though with considerably lower efficiency per glucose molecule. HIF also enhances the expression of lactate dehydrogenase A, facilitating the conversion of pyruvate to lactate [163]. Lactate has been found and is believed to contribute to wound healing in multiple chronic inflammatory disorders [164], including multiple sclerosis [165], pulmonary inflammation [166], and swollen joints [167-169]. On the other hand, elevated lactate concentrations cause acidosis and are thought to be harmful in certain inflammatory conditions as they lead to cell change and the creation of autoantigens [167]. Therefore, the identification of lactate in metabolomics investigations of sickness implies that the illness under investigation may have an inflammatory component, which may help with future research and treatment. It is also widely recognized that the ReA joint is hypoxic, which may contribute to metabolic changes that could enhance our understanding of the disease's etiology. A systemic inflammatory response modifies metabolism. Cachexia is the disease-related decrease of cellular mass and a sign of the significant correlation between metabolic processes and inflammation. TNFα was given the moniker “cachexin” after it was discovered to be involved in this procedure. Since TNFα is now widely recognized as a modulator of inflammatory reactions, it is instructive that inflammatory cytokines can have such a significant impact on cellular and metabolic functions. Chronic inflammation can lead to rheumatoid cachexia, characterized by the loss of muscle mass while fat mass is preserved [170]. Cachexia is usually associated with a low body mass index (BMI). Although muscle wasting is common in rheumatoid arthritis (RA), a low BMI is uncommon, as fat mass is often retained or even increased [171]. Consequently, individuals with RA may either exhibit the traditional low BMI cachexia, affecting 1-3% of RA patients [172], or more commonly, rheumatoid cachexia, which affects 10-20% of RA patients with controlled disease and 38% of those with active RA [173, 174]. Rheumatoid cachexia is thought to be driven by proinflammatory cytokines such as TNF, IL-1, and IL-6, which promote muscle loss. TNF, for example, enhances proteolysis through the ubiquitin-proteasome pathway. Research has shown that these cytokines inhibit anabolic resistance, the increase in muscle protein synthesis in response to feeding [175]. The extent of muscle atrophy in rheumatoid cachexia correlates with RA disease activity [175]. Additionally, both men and women with RA often have low or normal levels of free testosterone, which may contribute to rheumatoid cachexia. Population studies have linked low testosterone levels to obesity, particularly visceral fat accumulation. RA patients typically maintain normal diets but engage in less physical activity, leading to fat accumulation and a positive energy balance [176], which may be relevant in rheumatoid cachexia [175].

The intricate process of metabolism is impacted by both hereditary and environmental variables. Numerous studies have investigated specific metabolites in both patients and animal models of inflammation, as systemic inflammation significantly alters metabolism. As a result, we must examine multiple metabolites simultaneously, which is made possible by metabolomics, the systemic study of metabolites. By using this method, several metabolites associated with inflammatory disorders have been found, offering insights into the disease's causes as well as possible biomarkers.

### 6.2 Metabolomics and Inflammation

The significant systemic and localized metabolic changes described above are driven by inflammation and inflammatory cytokines, making it unsurprising that metabolomics has been used to study various inflammatory disorders. Metabolomics offers an integrated “top-down” view of the complex biochemical processes in living organisms by measuring global, dynamic metabolic responses through various analytical techniques [177]. The most used methods for metabolomic research are mass spectrometry (MS) and nuclear magnetic resonance spectroscopy (NMR) [178]. These techniques also assist in metabolite identification by providing structural details [179]. However, due to the vast diversity in the physical and chemical properties of metabolites, analyzing the entire metabolome with a single analytical method is practically a challenge. While metabolites, proteins, and genes are intricately linked in biological systems, metabolite levels do not always directly correlate with gene or protein expression. This underscores the need for additional measurements at the metabolite level and highlights the importance of metabolomics in studying gene-environment interactions [180]. While the goal of traditional genetics is to directly link DNA sequences to phenotypes, “-omic” technologies enable the focus to be shifted from a particular gene to the gene's real effects. A larger focus is being paid to the significance of assessing tiny molecular weight metabolites because it is not easy to directly correlate gene or protein profiles with the metabolic makeup. The technique known as “metabolomic profiling” has made it feasible to assess a subset of metabolites in biological materials, including tissues and bodily fluids, both quantitatively and qualitatively. Metabolomics, like other “-omic” research, seeks to study metabolite dynamics objectively and impartially [181].

Research on metabolism offers distinct advantages. As metabolic perturbations occur further down the biological information cascade from genes, transcripts, and proteins, they are often closer to the phenotype. The metabolome, being highly dynamic and capable of rapid changes within seconds, can serve as an early warning system for biological alterations. This makes it possible for metabolic disturbances to detect early changes in clinical systems long before the onset of disease symptoms, potentially reducing the need for more invasive interventions [182]. Metabolomic investigations follow a standard protocol [181-183]. After identifying a biological question and designing an experiment, samples are collected and prepared for analysis. The appropriate analytical techniques are then employed to gather data, which is subsequently pre-processed and analyzed before being interpreted in a biological context. Mass spectrometry (MS) and nuclear magnetic resonance (NMR) spectroscopy are commonly used in metabolomics research, each with its advantages and limitations. MS offers higher sensitivity, while NMR spectroscopy requires less sample preparation, is more reproducible, and can be used non-destructively [184, 185]. Advances in high-field clinical scanners and improved *in vivo* NMR spectroscopy techniques also hold promise for translating *ex vivo* NMR-based markers into *in vivo* diagnostics in the future.

### 6.3 The Metabolome

The term “metabolome,” introduced in 1998, refers to the complete set of small molecules in a biological sample. It combines “metabolite” and “chromosome” to highlight how metabolites are indirectly encoded by genes. Metabolomics, the study of the metabolome, aims to analyze these compounds, but unlike genomics and proteomics, it currently cannot identify the entire set of metabolites due to incomplete libraries and limitations in detecting low-concentration (less than 5µM) compounds [185].

The metabolome represents the relationship between an organism's environment and its DNA, making it a powerful indicator of its phenotype, which results from both environmental and genetic factors. Given that the etiology of ReA is influenced by both genetic and environmental factors, metabolomics could be a valuable tool in its study. The premise of metabolomics is that the levels of specific metabolites will change in response to disease-related alterations [186]. To be classified as a metabolite and included in the metabolome, a small molecule typically must have a molecular weight of less than 1500 Da. Examples include glycolipids, polysaccharides, and short peptides, which are distinct from proteins (studied in proteomics) and RNA or DNA (studied in genomics).

### 6.4 Metabolomics in Inflammatory Rheumatic Diseases

As mentioned earlier, a growing body of research highlights metabolomic changes in inflammatory diseases in both human and animal models. However, studies specifically exploring metabolomics in ReA are limited. Given the shared metabolic characteristics of inflammation across different conditions, research on metabolomics in other inflammatory diseases may provide valuable insights for studying ReA.

Using different biofluids, many metabolites have been found in other inflammatory illnesses; nevertheless, a higher energy requirement is a common theme with inflammation. Previous research has shown that people with ReA and RA require more energy, mostly because they produce more TNF [174, 187]. Investigations on inflammatory lung disease [166], OA [188], and multiple sclerosis [189] have indicated a decrease in glucose. In contrast, investigations on inflammatory eye disease [190] and inflammatory lung disease [166] have revealed an increase in lactate. The joint has been linked to oxidative stress [191] and has been shown to have a hypoxic environment [192, 193] in RA investigations. In mice, xanthine has been demonstrated to differentiate RA from controls [191], and similar results have been obtained in inflammatory bowel disease [194].

Although various metabolites have been associated with a range of inflammatory diseases, a metabolomic study focused on identifying unique metabolic profiles in arthritis patients could significantly enhance our understanding of the etiology and pathophysiology of ReA. Specific metabolites have been linked to particular diseases, and when combined with laboratory tests, metabolomics can serve as a powerful tool in “systems medicine,” allowing for the consistent and precise identification of metabolites from minimal biological samples.

#### 6.4.1 Rheumatoid Arthritis (RA)

The metabolism of the inflammatory synovium can be altered due to various factors, such as increased joint metabolism or impaired vascularity. Hyaluronic acid, an essential component of the proteoglycan aggregate in articular cartilage, is crucial for maintaining the functional integrity of the extracellular matrix. Reactive oxygen radical species in RA depolymerized SF hyaluronate [195], and neither normal nor inflamed SF had any hyaluronidase activity. Reactive oxygen species production is crucial to the damage caused by synovial hypoxia reperfusion [196]. This happens when the synovial capillary perfusion pressure is exceeded by the raised intra-articular pressure during exercise, which results in reduced blood flow [155].

The NMR profiles of RA-matched serum and SF samples were examined in 1993 by the London Hospital Medical College's Inflammation Research Group [192]. When comparing the NMR-based metabolic profiles of the SF to the matched serum samples, it was found that the SF had much higher amounts of lactate and lower levels of glucose. These metabolic changes align with the hypoxic conditions observed in rheumatoid joints [192]. Compared to corresponding blood samples, synovial fluid (SF) samples from both RA and control groups exhibited lower levels of chylomicrons and triglycerides associated with very low-density lipoprotein. Additionally, SF samples had higher concentrations of ketone bodies compared to matching serum samples. These results suggest that, despite the hypoxic environment, the intra-articular space relies more heavily on lipids for energy [192, 193]. Furthermore, a metabolite biomarker pattern associated with RA has been identified in mouse serum [191]. Using NMR-based metabolic profiling, researchers found that uracil, xanthine, and glycine could distinguish arthritic mice from controls [191]. The detected metabolites point to a potential link between oxidative stress and a significant impact on nucleic acid metabolism in RA.

In more recent times, RA patient plasma has been studied by a Danish study team [197]. In contrast to healthy controls and RA in remission, they discovered that RA patients had different metabolic profiles. The acetylated glycoprotein, cholesterol, lactate, and lipids were the identified metabolites that allowed the active RA patients to be distinguished from the healthy controls. Indirectly reflecting active inflammation, the lactate levels revealed oxidative damage [198]. This suggests that metabolomics could serve as a valuable tool for assessing disease severity, potentially differentiating patients in true remission from those with low disease activity. It also indicates that significant metabolic changes during inflammation can be detected in peripheral blood.

Using synovial fluid, a recent clinical investigation examined the lipid profiles of RA patients [199]. They discovered nearly seventy distinct lipid components with pro-resolving and anti-inflammatory characteristics. Combining metabolomics with traditional Chinese medicine has also been utilized to further classify various RA patient types [200]. They analyzed the metabolomics of plasma and urine samples alongside symptom profiles of RA patients. By combining Cold and Heat type methodologies with traditional Chinese medicine, they identified notable biochemical differences among RA subgroups, suggesting various disease mechanisms and potential for tailored treatments. Additionally, metabolic studies have shown the ability to predict RA patients' responses to specific treatments [201]. For example, a study of 38 active RA patients found significant differences in metabolite levels between those who responded to methotrexate and those who did not [201].

#### 6.4.2 Ankylosing Spondylitis (AS)

A subtype of inflammatory arthritis known as spondyloarthritis includes, most famously, ankylosing spondylitis (AS). Unfortunately, most people with AS must wait ten years from the onset of symptoms before a diagnosis is made, resulting in a significant delay in diagnosis. However, MRI (magnetic resonance imaging) has proven to be a viable diagnostic method based on modifications in imaging data. While not all patients acquire AS, these individuals may be considered to have early AS. A group of AS patients and controls may be distinguished by Gao *et al*. [202]. This study also revealed the downregulation of vitamin D serum metabolites, including 23S,25R-hydroxyvitamin D3 and 23-peroxylacetone. While further research is needed, this metabolite, produced after the kidney converts 25(OH)D3, may suggest that disease states impact vitamin D3 metabolism [203].

#### 6.4.3 Systemic Lupus Erythematosus (SLE)

Using serum samples, Ouyang *et al*. utilized ^1^H NMR spectroscopy to differentiate between patients with systemic lupus erythematosus (SLE), rheumatoid arthritis (RA), and healthy controls. They found elevated concentrations of N-acetyl glycoprotein in SLE samples, alongside increased levels of low-density lipoprotein (LDL) and very low-density lipoprotein (VLDL), and decreased levels of high-density lipoprotein (HDL), valine, tyrosine, phenylalanine, lysine, isoleucine, histidine, glutamine, alanine, citrate, creatinine, creatine, and pyruvate compared to controls. This “lupus pattern” reflects elevated LDL and VLDL with reduced HDL. Given that polyunsaturated fatty acids are precursors to inflammatory mediators, these changes may be linked to the inflammatory process [204]. Lower citrate and pyruvate levels might indicate increased energy demands in inflammatory conditions. Additionally, SLE significantly increases lipid peroxidation damage, a cardiovascular risk factor damage [205, 206]. These findings highlight the potential of metabolomic biomarkers for diagnosis and provide insight into SLE pathoaetiology. PCA and partial least squares discriminant analysis models, as shown by Dai *et al*., can distinguish SLE patients from healthy controls with 97.1% specificity and 60.9% sensitivity [204].

### 6.5 Approaches to Metabolomics

Metabolomics aims to systematically identify and measure metabolites from biological samples. These small molecules, resulting from complex biological processes within specific cells, tissues, or organs, are valuable for studying disease mechanisms. Analyzing metabolites—such as lipids, amino acids, peptides, nucleic acids, and organic acids—presents challenges due to their diverse, low molecular weight structures, generally under 1500 Da [207]. Recent advancements in MS and NMR-based analytical platforms have facilitated the separation, characterization, detection, and quantification of these chemically varied structures [208, 209]. Unlike transcriptomics and proteomics, genomic data cannot determine the molecular identity of metabolites [210]. Therefore, sophisticated tools like NMR and MS spectroscopy are essential for metabolite identification and quantification (Table **1**). Each technology offers distinct advantages and limitations, and the choice of method typically involves a trade-off between speed, selectivity, and sensitivity, depending on the study's specific goals [211-221].

#### 6.5.1 Mass Spectrometry

The MS method provides a strong balance of selectivity and sensitivity (Table **1**). Modern MS has exceptional sensitivity and allows for the detection and measurement of various primary and secondary metabolites at very low levels. It also delivers highly detailed chemical information directly related to the compounds being analyzed. These advantages make MS a valuable tool in metabolomics [222].

#### 6.5.2 NMR Spectroscopy

Although NMR spectroscopy was first developed in the 1940s, its sensitivity has improved with advances in magnetic field strength [178]. NMR is non-destructive, highly selective, and widely recognized as the gold standard for elucidating metabolite structures, though it is less sensitive than MS. Unlike other detection techniques, NMR does not rely on separating analytes, allowing for sample recovery and further analysis [180]. NMR can simultaneously evaluate a wide range of small molecule metabolites, making it almost a “universal detector.” Its key advantages include high repeatability and minimal sample preparation [223]. Additionally, NMR is considered a more reliable method for determining molecule concentrations compared to MS [178].

### 6.6 NMR Based Metabolomics

In metabolomics, nuclear magnetic resonance (NMR) spectroscopy is a powerful analytical method commonly used for the qualitative and quantitative analysis of low molecular weight metabolites in complex biological materials. NMR was discovered in 1946 by two separate teams: F. Bloch, W. Hansen, and M. Packard at Stanford University, and E. M. Purcell, R. V. Pound, and H. C. Torrey at Harvard University. The discovery revealed that magnetic nuclei, such as ^1^H, ^13^C, and ^31^P, could absorb radio frequency radiation when exposed to a magnetic field. Since then, NMR has been applied to kinetic and structural studies across solids, liquids, and gases, earning six Nobel Prizes in Chemistry.

### 6.7 NMR Spectroscopy

NMR spectroscopy is a versatile analytical method used to identify and measure a wide range of biological metabolites. It applies to nuclei with an intrinsic magnetic moment and non-zero spin. Commonly studied spin ½ nuclei include ^1^H (the most frequently used in NMR), ^13^C, ^19^F, and ^31^P. In contrast, isotopes like ^12^C and ^16^O, with an even number of protons and neutrons, have paired spins that cancel out and cannot be studied with NMR spectroscopy. Protons (^1^H), abundant in organic and biological samples, are often used in magnetic resonance studies, while phosphorus NMR (^31^P) is particularly useful for researching phospholipids and energy metabolism [224].

Without an applied magnetic field (B_0_), nuclear spins are randomly oriented. When exposed to a magnetic field, nuclei align either parallel or antiparallel to the field, with the parallel alignment having lower energy. The energy difference (ΔE) between these states is proportional to the strength of B_0_. An external radio frequency (RF) pulse can excite spins from a lower energy state to a higher one. As spins return to their lower energy state, they emit energy as radio waves, which are detected as the MR signal [225, 226].

The MR spectrum plots the intensity of the magnetic resonance signal against the frequency of the magnetic field. Spin relaxation processes, which occur as spins return to equilibrium, are known as longitudinal (T1) and transverse (T2) relaxation. T1 relaxation involves the transfer of net energy to the environment; smaller molecules typically have a longer T1, while larger molecules, such as proteins and lipids, have a shorter T1. T2 relaxation, on the other hand, is determined by the dephasing of individual magnetic moments and can be influenced by factors such as temperature, viscosity, free water content, the presence of paramagnetic atoms, and field homogeneity.

The T1 and T2 values are crucial for selecting appropriate NMR acquisition techniques. To obtain accurate relative signal intensities, a recycle delay of at least five times the longest T1 is needed to ensure that all nuclei return to equilibrium before the next excitation. Additionally, molecules with short T2 values can help filter out signals from larger macromolecules like lipids.

Chemical shifts, small variations in resonance frequency caused by the shielding effect of the electron cloud, reflect slight changes in the local magnetic field experienced by the nucleus. These shifts, measured in parts per million relative to a common reference frequency, provide detailed information about the molecular environment. Stronger magnetic fields enhance spectral resolution, allowing for precise differentiation of overlapping resonances, such as those from glycerophosphocholine (GPC), phosphocholine (PCho), and free choline, thereby improving quantification accuracy [227].

## 7 LIMITATIONS OF METABOLOMICS IN INFLAMMATORY RHEUMATIC DISEASES

Metabolomics has emerged as a vital field in understanding inflammatory rheumatic diseases such as rheumatoid arthritis (RA), ankylosing spondylitis (AS), and systemic lupus erythematosus (SLE). By profiling metabolites in biological samples, researchers aim to uncover biomarkers associated with these diseases and elucidate their underlying pathophysiological mechanisms. However, despite its potential, several limitations hinder the effective application of metabolomics in clinical and research settings.

### 7.1 Challenges in Metabolomic Data Interpretation

One of the significant limitations of metabolomics in the context of inflammatory rheumatic diseases is the intrinsic complexity of biological systems [228]. Various environmental factors, disease states, and genetic variations contribute to a heterogeneous landscape of metabolites present in biological samples [229]. This variability complicates the task of establishing clear cause-and-effect relationships between specific metabolites and disease pathology [228]. Furthermore, the data generated from metabolomics studies often require advanced analytical methods to differentiate biologically relevant signals from noise, making data analysis and validation particularly challenging [229].

### 7.2 Standardization Issues

Standardization poses another major hurdle for metabolomics research within this domain [230]. The absence of universally accepted protocols for sample collection, storage, and analysis can lead to significant discrepancies in metabolite concentrations between studies, complicating cross-study comparisons and replicability of results [228]. Moreover, the need for high-throughput technologies and specialized equipment is often a barrier, as many clinical laboratories lack such resources. This limitation restricts the broader adoption of metabolomics approaches in routine clinical practice, ultimately affecting the translation of research findings to patient care [228].

### 7.3 Specificity and Sensitivity Concerns

Metabolomics faces notable limitations regarding the specificity and sensitivity of biomarkers in inflammatory rheumatic diseases [228]. While some metabolites may be highlighted as potential disease markers, their concentrations can be skewed by various factors unrelated to the disease—such as diet, lifestyle, and coexisting medical conditions [228]. This variability can lead to false-positive or false-negative outcomes, casting doubt on the clinical utility of these biomarkers for accurate diagnosis or monitoring of the diseases [228]. Without robust validation, the effectiveness of metabolomic biomarkers remains uncertain in the clinical context.

### 7.4 Integrative Approaches Necessary

To enhance the understanding of disease mechanisms in RA, AS, and SLE, integrative approaches that combine metabolomics with genomics and proteomics are often essential [228]. This multifaceted integration can provide a more comprehensive view of the biological pathways involved. However, combining data from multiple omics platforms introduces additional complexity in data interpretation and necessitates sophisticated computational skills that may not be available in all research environments [228]. Such integrative strategies are crucial for accurately correlating metabolomic findings with the underlying biological processes of these diseases.

## 8 CURRENT CLINICAL TREATMENTS AND CHALLENGES OF REACTIVE ARTHRITIS

Conventional therapy generally includes non-steroidal anti-inflammatory drugs (NSAIDs) to alleviate joint pain and inflammation. For cases resistant to NSAIDs, disease-modifying antirheumatic drugs (DMARDs), particularly biologics, are often employed [231]. These biological agents target specific pathways involved in the inflammatory process. However, the evidence supporting their efficacy for refractory cases of ReA remains insufficient [232].

### 8.1 Non-Steroidal Anti-Inflammatory Drugs (NSAIDs)

NSAIDs are often the first line of treatment for ReA due to their efficacy in reducing inflammation and providing symptomatic relief [233]. Studies have shown that most patients with ReA respond favorably to NSAID therapy; indeed, over 50% of patients experience a self-limited course of the disease, lasting two to six months, and NSAIDs contribute significantly to this positive outcome [233]. They can effectively manage symptoms such as joint pain and swelling, which are hallmark features of the condition [234]. A variety of NSAIDs may be prescribed depending on individual patient needs and responses. Common choices include Ibuprofen, Naproxen and Indomethacin. Each type of NSAID comes with its own benefits and potential side effects, including gastrointestinal issues, which necessitate careful patient monitoring [235].

### 8.2 Disease-Modifying Anti-Rheumatic Drugs (DMARDs)

Disease-modifying anti-rheumatic drugs (DMARDs) are essential in the treatment of Reactive Arthritis (ReA), particularly in cases with chronic or severe presentations where inflammatory symptoms persist despite initial therapy [236]. DMARDs play a critical role in modulating the immune response and preventing long-term joint damage associated with chronic inflammation. Studies show that treatment with DMARDs can lead to significant improvements in symptom control and joint function in patients with refractory ReA. For instance, a long-term effectiveness study found that patients with inadequate responses to conventional NSAIDs showed outstanding outcomes when transitioning to csDMARDs, particularly methotrexate [237]. In refractory cases, DMARDs not only alleviate symptoms but also help in achieving remission of joint inflammation, thus preventing chronic complications [238].

#### 8.2.1 Corticosteroids

Corticoids have shown efficacy in controlling severe cases of reactive arthritis, assisting in symptom relief and reducing the progression of joint damage. They can be particularly beneficial in managing severe inflammation associated with reactive arthritis, especially when Non-steroidal anti-inflammatory drugs are ineffective or non-tolerated [239]. These may be utilized for short-term relief during active flare-ups [240].

#### 8.2.2 Challenges in Treatment

The primary challenge in treating reactive arthritis lies in its heterogeneity [241]. Patients exhibit a wide spectrum of responses to traditional therapies, making it difficult to standardize treatment protocols. Moreover, while antibiotic therapy is crucial for treating the initial infection, its effectiveness in addressing arthritis is less clear. Standard antibiotics often do not provide an effective cure for ReA symptoms, necessitating advanced treatment protocols [242].

#### 8.2.3 Innovative Therapeutic Strategies

Recent research is exploring novel treatment avenues, notably the potential application of vagus nerve stimulation as an adjunctive therapy [243]. This non-invasive approach has shown promise in reducing disease severity in rheumatoid arthritis and could extend applications to reactive arthritis. Additionally, combination antibiotic therapy has emerged as a viable option for addressing the underlying infectious causes of ReA, particularly when traditional treatments have failed [242].

## 9 FUTURE DIRECTIONS

There is a pressing need for further research initiatives to better understand the pathophysiological mechanisms behind reactive arthritis. Enhanced understanding of host-pathogen interactions may inform more effective therapeutic strategies tailored to individual patient profiles. Ongoing clinical studies will be crucial in assessing the long-term efficacy and safety of emerging therapies, as well as in establishing guidelines that integrate both symptom relief and disease management comprehensively.

In summary, while current treatments for reactive arthritis provide a foundation for managing symptoms, there are significant challenges due to its variable nature and the limited efficacy of traditional therapies. The exploration of innovative approaches and comprehensive research will be vital to improving outcomes for affected patients.

## CONCLUSION

Reactive arthritis (ReA) is a multifaceted immune-mediated condition influenced by the interplay of cytokines and systemic metabolic alterations. The balance between Th1 and Th2 cytokines, including the antibacterial activities of TNF-α and IFN-γ and the humoral focus of IL-4, IL-5, IL-9, and IL-13, plays a critical role in shaping immune responses during ReA pathogenesis. However, the mechanisms underlying T helper cell differentiation and the resultant cytokine secretion profiles remain incompletely understood, with factors such as the inflammatory milieu, IL-12 levels, antigen-presenting cell variability, and antigen dosage emerging as key contributors. ReA also impacts systemic metabolism through complex interactions between genetic and environmental factors. Given that both lifestyle and genetic predispositions influence metabolic processes, analyzing metabolites can provide early biomarkers of inflammatory disease. NMR metabolomics, in particular, is a promising tool for detecting subtle metabolic changes indicative of ReA's onset, offering a pathway to earlier diagnosis. This review underscores the potential of metabolomics to bridge gaps in our understanding of ReA pathogenesis by elucidating the metabolic pathways that interact with immune mechanisms. By integrating metabolic and immunological insights, future research holds promise for refining diagnostic techniques, improving therapeutic strategies, and advancing personalized management approaches for ReA and related seronegative spondyloarthropathies.

## Figures and Tables

**Fig. (2):**
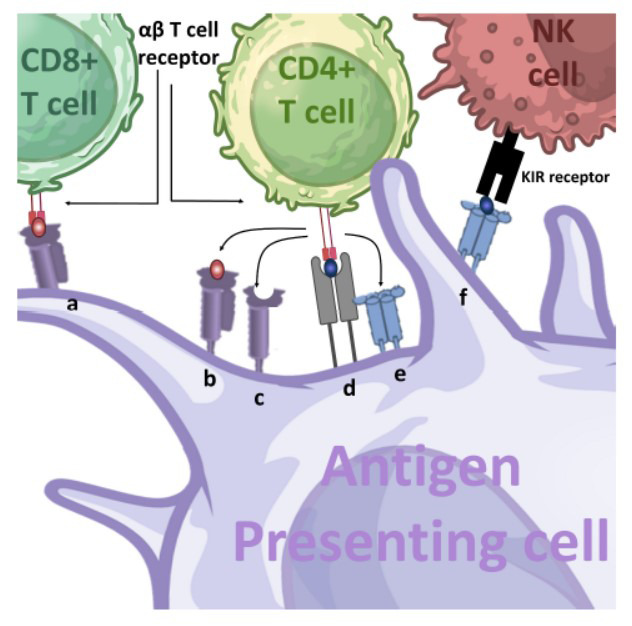
Describe many potential discoveries about the role of HLA B-27 molecules in the pathophysiology of ReA: (**a**) HLA-B27/β2m and peptide complex recognized by CD8^+^ T cells. (**b**) HLA-B27/β2m and peptide complex recognized by CD4^+^ T cells. (**c**) Free HLA-B27 heavy chain interacting with CD4^+^ T cells. (**d**) HLA class II (DR, DQ, DP) presenting HLA-B27-restricted peptide to CD4^+^ T cells. (**e**) HLA-B27 homodimers interacting with CD4^+^ T cells. (**f**) HLA-B27 homodimers recognized by receptors on NK cells.

**Table 1:** Approaches for metabolite identification and quantification.

**Source**	**Treatment Usage**	**Platform**	**Metabolites**	**Refs.**
Plasma	Methotrexate	MS	Plasma levels of nornicotine, N-methyl isoleucine, and 2,3-dihydroxy butanoic acid enhanced the capacity to accurately predict methotrexate responders.	[211]
Serum	DMARDS: MTX or leflunomide; bDMARDS: TNF	HPLC-MS/MS	**Threonine:** Differentiation of RA patients receiving MTX/leflunomide compared to those treated with infliximab/adalimumab/etanercept/tocilizumab and infliximab/adalimumab/etanercept/tocilizumab-prednisolone/NSAID **Tryptophan:** differentiated RA patients treated with methotrexate/leflunomide *versus* infliximab/adalimumab/etanercept/tocilizumab.	[212]
Serum	Etanercept	1H NMR	Levels of isoleucine, leucine, valine, alanine, glutamine, tyrosine and glucose rise and 3-hydroxybutyrate levels drop in Etanercept excellent responders.	[213]
Blood	Infliximab, abatacept or etanercept	RP-UHPLC ESI-QTOF-MS	Two distinct metabolic profiles differentiate good responders from non-responders: Carbohydrate derivatives include D-glucose, D-fructose, sucrose, and maltose.	[214]
Plasma	Tocilizumab	1H-NMR	The concentrations of 3-hydroxybutyrate and phenylalanine enhanced the specificity of predicting TCZ responders.	[215]
Serum	Rituximab	NMR-MS	Responders showed lower levels of phosphatidylethanolamines, phosphatidyserines, and phosphatidylglycerols, while 37 lipids differed between responders and non-responders.	[216]
Serum	TNFi	CE-TOFMS	Betonicine, glycerol 3-phosphate, N-acetylalanine, hexanoic acid, and taurine have been linked to TNFi responses in RA. Abatacept is associated with citric, quinic, and 3-aminobutyric acids.	[217]
Serum	Tocilizumab	MS	Changes in the metabolism of arachidonic acid	[218]
Serum	Etanercept/adalimumab	1H NMR	3-hydroxyisobutyrate, lysine, L5, acetoacetate, creatine, GPC+APC, histidine, and phenylalanine exhibited elevated levels in rheumatoid arthritis, while leucine, acetate, betaine, and formate showed decreased levels.	[219]
Serum	Tofacitinib/baricitinib	1H-NMR	DHA levels of omega-3 fatty acids were elevated in patients treated with JAK inhibitors. DHA was linked to reductions in pain.	[220]
Serum	GC	LC-MS/MS	Increased levels of lysophosphatidylcholines and lysophosphatidylethanolamines in females.	[221]

## LIST OF ABBREVIATIONS

ReA: Reactive Arthritis
SSA: Seronegative Spondyloarthropathy
uSpA: Undifferentiated Spondyloarthritis
AS: Ankylosing Spondylitis
PsA: Psoriatic Arthritis
IBD-A: Inflammatory Bowel Disease Associated Arthritis
HLA-B27: Human Leukocyte Antigen B27
TNF-α: Tumor Necrosis Factor Alpha
IFN-γ: Interferon Gamma
IL: Interleukin
ESR: Erythrocyte Sedimentation Rate
CRP: C-Reactive Protein
ANA: Antinuclear Antibody
RF: Rheumatoid Factors
LCR: Ligase Chain Reaction
PCR: Polymerase Chain Reaction
SFMCs: Synovial Fluid Mononuclear Cells
ELISA: Enzyme-Linked Immunosorbent Assay
NF-κB: Nuclear Factor Kappa B
APCs: Antigen-Presenting Cells
MLN: Mesenteric Lymph Node
HIF: Hypoxia-Inducible Factor
RA: Rheumatoid Arthritis
VLDL: Very Low-Density Lipoprotein
LDL: Low-Density Lipoprotein
HDL: High-Density Lipoprotein
SLE: Systemic Lupus Erythematosus
NMR: Nuclear Magnetic Resonance
MS: Mass Spectrometry
DMARDs: Disease-Modifying Antirheumatic Drugs
bDMARDs: Biological Disease-Modifying Antirheumatic Drugs
GC: Gas Chromatography
LC-MS/MS: Liquid Chromatography Tandem Mass Spectrometry
CE-TOFMS: Capillary Electrophoresis-Time-of-Flight Mass Spectrometry
DHA: Docosahexaenoic Acid
JAK: Janus Kinase
UHPLC-HRMS: Ultra-High Performance Liquid Chromatography-High-Resolution Mass Spectrometry
NSAIDs: Non-Steroidal Anti-Inflammatory Drugs
SF: Synovial Fluid
GPC: Glycerophosphocholine
PCho: Phosphocholine
CpG: Cytosine-phosphate-Guanine
LPS: Lipopolysaccharide
TGF-β: Transforming Growth Factor Beta
β2m: Beta-2 Microglobulin
MHC: Major Histocompatibility Complex
CD: Cluster Differentiation
Th: T helper
TAP: Transporter Associated with Antigen Processing
HSP: Heat Shock Protein
KIR: Killer Immunoglobin Receptor
UPR: Unfolded Protein Response
AA: Arachidonic Acid
EPA: Eicosapentaenoic Acid
ATP: Adenosine Triphosphate
